# Scarlatina and the Puerperal State

**Published:** 1863-05

**Authors:** A. Clemens


### SCARLATINA AND THE PUERPERAL STATE.

Every epidemic of scarlatina is accompanied by an increased number of abortions and premature deliveries.

The appearance of scarlet fever during child-bed is a dangerous complication, on account of this specific relation of the contagion to the womb. Halm described, in 1837, the scarlatina puerperalis as differing from scarlet fever in not being contagious, appearing within three or four days after delivery, frequently not affecting the mucous membranes at all, and showing no regularity in regard to the fever and eruption. The fever comes suddenly, with a well-marked chill, and a very quick, full, hard pulse. Slight pains in the womb disappear with the coming eruption, which usually covers rapidly the greater part of the body, frequently on the second day of sickness, without mitigation of the fever. This disappears, in favorable cases, with eruption on the third or fourth day, when desquamation follows immediately. Without any apparent cause, this may result in peritonitis, splenitis, dropsy, or pleuritis, wlth extensive exudation. Where no desquamation takes place, the fever goes on and mania supervenes, soon ending in death. Headache during the eruption, and pleuritis and peritonitis subsequently, are very unfavorable symptoms. The treatment must be antiphlogistic, with small doses of calomel. Ablutions with warm water, mineral acids, cold applications, leeches, according to circumstances.—*A. Clemens.—Am. Monthly, Trans.*

### PHYSICAL DISEASE IN INSANITY.

In every disease of the mind, mental phenomena must necessarily have physiological facts for their basis, and even as a condition of their existence. What symptoms are we, then, obliged to find and describe in the so-called moral insanity, or diastrephia? I answer—both moral and physical: the first relating tó a perversion of the human will and instincts; the second, the somatical facts, the symptoms perceptible to our senses. These points settled, all the other evidences support, always, the fundamental inquiries. Out of that, physicians do not want to venture in vague theories; and for them, *what is not disease in diastrephia is crime.—Parigot.*

Ovariotomy in London.—Ovaritomy seems to be prevailing epidemically in London. The number of the *Lancet* for Dec. 20th last, contains notices of 75 cases of this operation. Of these, 43 are said to have recovered and 26 were fatal; in three cases the operation was commenced but not completed, and in three an exploratory incision was made in aid of diagnosis, in one with a fatal result.

In connection with this subject we must not omit to notice the *modesty* of the editor of the *Lancet*, who, in an editorial in the number just quoted, claims Ovariotomy as *a triumph of British Surgery!* He observes: "This noble operation —for the saving of 50 per cent. of poor creatures from absolutely impending death surely deserves the epithet—is essentially a triumph of British Surgery."—*Med. News.*

